# A good investment: longer-term cost savings of sensitive parenting in childhood

**DOI:** 10.1192/j.eurpsy.2022.1074

**Published:** 2022-09-01

**Authors:** C. Bachmann, J. Beecham, T. O’Connor, J. Briskman, S. Scott

**Affiliations:** 1 Universitätsklinikum Ulm, Dept. Of Child & Adolescent Psychiatry, Ulm, Germany; 2 London Schoolof Economics and Political Science, Pssru, Canterbury, United Kingdom; 3 University of Rochester, Dept. Of Psychiatry, Rochester, United States of America; 4 Institute of Psychiatry, Psychology and Neuroscience, Dept. Of Child & Adolescent Psychiatry, London, United Kingdom

**Keywords:** costs, sensitive responding, Children, parenting

## Abstract

**Introduction:**

Good quality parenting in early childhood is reliably associated with positive mental and physical health over the lifespan.

**Objectives:**

The hypothesis that early parenting quality has significant long-term financial benefits has not been previously tested.

**Methods:**

Design: Longitudinal study with follow-up from 2012 to 2016*;* UK multicentre study cohort. *Participants:* 174 young people drawn from 2 samples, one at moderate risk of poor outcomes and one at high risk, assessed aged 4–6 years then followed up in early adolescence (mean age 12.1 years). *Measures* **:** The primary outcome was total costs: health, social care, extra school support, out-of-home placements and family-born expenditure, determined through semistructured economic interviews. Early parenting quality was independently assessed through direct observation of parent–child interaction.

**Results:**

Costs were lower for youths exposed to more sensitive parenting (most sensitive quartile mean £1,619, least sensitive quartile mean £21,763; *p* < .001). Costs were spread across personal family expenditure and education, health, social and justice services. The cost difference remained significant after controlling for several potential confounders. These included demographic variables (family poverty, parental education); exposure to child abuse; and child/young person variables including level of antisocial behaviour in both childhood and adolescence, IQ and attachment security.

**Conclusions:**

This study is the first showing that more sensitive early parental care predicts lower costs to society many years later, independent of poverty, child and youth antisocial behaviour levels and IQ. The findings provide novel evidence for the public health impact of early caregiving quality and likely financial benefits of improving it.

**Disclosure:**

No significant relationships.

